# Ecological drivers of *Mycobacterium avium* subsp. *paratuberculosis* detection in mongoose (*Herpestes ichneumon*) using IS*900* as proxy

**DOI:** 10.1038/s41598-020-57679-3

**Published:** 2020-01-21

**Authors:** Mónica V. Cunha, Luís Miguel Rosalino, Célia Leão, Victor Bandeira, Carlos Fonseca, Ana Botelho, Ana C. Reis

**Affiliations:** 1 INIAV, IP- National Institute for Agrarian and Veterinary Research, Av. da República, Quinta do Marquês, 2780 -157 Oeiras, Portugal; 2 Centre for Ecology, Evolution and Environmental Changes (cE3c), Faculdade de Ciências da Universidade de Lisboa, Campo Grande, 1749-016 Lisboa Portugal; 3 Biosystems & Integrative Sciences Institute (BioISI), Faculdade de Ciências da Universidade de Lisboa, Campo Grande, 1749-016 Lisboa Portugal; 4 CESAM & Departamento de Biologia, Universidade de Aveiro, Aveiro, Portugal

**Keywords:** Pathogens, Ecological epidemiology

## Abstract

*Mycobacterium avium* subsp. *paratuberculosis* (*MAP*) is the etiological agent of Johne’s disease or paratuberculosis, a chronic infection affecting domestic ruminants worldwide. Despite sporadic reports of *MAP* occurrence in non-ruminants, information on the risk factors predisposing for infection is still scarce and evidence of transmission paths linking the livestock-wildlife-environment interfaces also remains lacking. In this study, we predicted that environmental, host-related, land use and human driven disturbance factors would modulate carnivore exposure to *MAP*. To test these hypotheses, we performed a retrospective survey, based on microbiological and molecular methods, in mainland Portugal including five sympatric species from the *Herpestidae*, *Canidae*, *Viverridae*, and *Mustelidae* families (n = 202) and examined 16 variables as putative predictors of *MAP* occurrence. Molecular evidence of *MAP* using IS*900* as proxy was demonstrated in 7.43% (95%CI: 4.55–11.9) of surveyed carnivores, the highest proportions being registered for red fox (*Vulpes vulpes*) (10%; 95%CI: 4.0–23) and Egyptian mongoose (*Herpestes ichneumon*) (6.0%; 95%CI: 3.2–11). We demonstrate that important species of the Mediterranean carnivore guild, such as stone marten (*Martes foina*) and common genet (*Genetta genetta*), may also be exposed to *MAP*, being this the first time that occurrence in genet is reported. The high proportion of DNA-positive specimens, concurrent with the apparent lack of gastro-enteric lesions and molecular confirmation of IS*900* in feces, argue for the presence of subclinical carriers that occasionally shed bacteria, potentially aiding as source of infection to susceptible species and possibly contributing for environmental contamination. Achievement of *MAP* isolation would prove beyond any doubt that *MAP* is present in this wildlife population. Ecological modelling results suggested that the probability of *MAP* infection using IS*900* as proxy in mongoose is positively associated with higher altitude and temperature stability, as well as with lower annual rainfall. Density of livestock farms was found not to be a significant predictor, which may indicate that the livestock-wildlife interface is probably not important as an infection route for mongoose.

## Introduction

*Mycobacterium avium* subspecies *paratuberculosis* (*MAP*) is a member of the *Mycobacterium avium* complex (MAC) that also includes *M. avium* subsp. *avium*, *M. avium* subsp. *silvaticum*, and *M. avium* subsp. *hominissuis*. This group of fastidious, phenotypically diverse, bacteria has specific pathogenicity and host range features, worldwide distribution and presumed zoonotic potential^1–3^.

*MAP* is an infectious enteric pathogen that causes paratuberculosis or Johne’s disease, a chronic infection of the gastrointestinal tract that affects ruminants worldwide^4^. According to the OIE Worldwide Animal Health Information Database (WAHID interface), 58 out of 241 countries reported *MAP* infection during 2017. While not all countries report infection, paratuberculosis is a communicable disease that is widespread and represents a major problem for animal health, causing substantial economic losses that differ according to the production system, herd size, or herd management type^4^. Different figures have been advanced for both dairy and beef cattle and these may vary from country to country (reviewed in Garcia and Shalloo, 2015^5^). In the USA alone, costs have been estimated from $200 to $1,500 million^6–8^. Financial losses are related with decreased milk productivity, reduced feed conversion efficiency and slaughter value, limitations on animal trade and transactions of animal products, and increased costs related with premature culling and veterinary care^5^.

Ruminants appear to be the preferred or natural host for *MAP*, primarily domestic ruminants such as cattle, sheep and goat, but the disease in wild ruminants is also well documented, including red deer (*Cervus elaphus*), roe-deer (*Capreolus capreolus*), fallow deer (*Dama dama*), white-tailed deer (*Odocoileus virginianus*), alpine ibex (*Capra ibex*) and riverine buffalo (*Bubalus bubalis*)^9–14^.

In non-ruminant animals, *MAP* was first detected in wild rabbits (*Oryctolagus cuniculus*) in Scotland^15,16^. After this finding, the research for the presence of this pathogen was extended to other wildlife species, firstly in areas with previous history of paratuberculosis in livestock, which allowed the detection of *MAP* in a very broad host range, including brown bear (*Ursus arctos*), raccoon (*Procyon lotor*), opossum (*Didelphis virginiana*), coyote (*Canis latrans*), red fox (*Vulpes vulpes*), stoat (*Mustela erminea*), weasel (*Mustela nivalis*), wood mouse (*Apodemus sylvaticus*), European badger (*Meles meles*), hare (*Lepus europaeus*) and jackdaw (*Corvus monedula*)^14,17–21^.

Disease progression and clinical signs are best characterized in domestic ruminants, where the pathogen typically enters the host by the oral route and then, eventually, will cause a progressively chronic inflammatory state with the presence of granulomatous lesions in the small and large intestines and in the mesenteric lymph nodes, leading to progressive weight loss, diarrhea and decreased milk production^22^. *MAP* excretion occurs mainly in feces but, in advanced stages of infection, the pathogen may also be found in colostrum and milk, saliva, uterine fluid and semen, and cause fetal infections *in utero*^22–25^. As such, the main route of intra- and interspecies transmission is considered fecal-oral by direct ingestion of *MAP*-contaminated feces, infected colostrum or milk or, indirectly, via *MAP*-contaminated water and/or feed (livestock feed, vegetation and/or infected prey)^26,27^.

**Figure 1:**
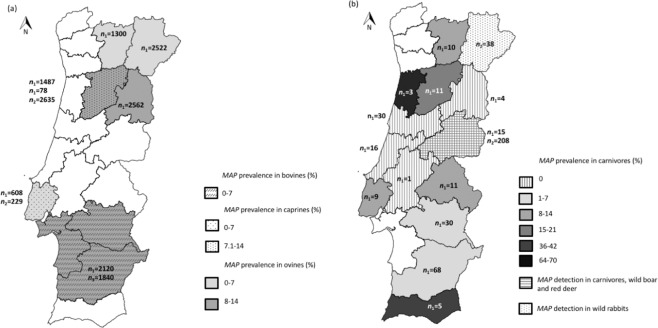
**(a)** Regional prevalence of *MAP* in mainland Portugal, showing districts as sampling unit at the administrative level. *MAP* distribution and prevalence intervals per district, in ovine, caprine and bovine subpopulations are evidenced in grey scale, dots and dashes, respectively, as indicated. Data represented was compiled from previous studies^31–33^. *n* – number of animals serologically tested; *n*_1_ - ovine; *n*_2_ - caprine and *n*_3_ – bovine; **(b)** *MAP* distribution in the wild: carnivore species (grey shade; data from this study), rabbits (dots^77^) and carnivores, wild boar and red deer from Central East/Castelo Branco district (horizontal lines^36–38^). *n*_1_ - number of animals tested in this study; *n*_2_ *-* number of animals tested in previous studies. The proportion of exposed carnivores determined in the current study is shown as intervals.

In Portugal, paratuberculosis was first reported in cattle from Lisboa region by Petisca and colleagues, in 1961^28^. Since then, geographically-limited studies, circumscribed to the North, Alentejo region and Lisbon, have tried to assess its occurrence, mainly in small ruminants^29–33^. Reports refer mainly to serological or molecular evidences rather than to clinical or anatomo-histological data^29–34^, suggesting that probably this disease is under-diagnosed. These studies, spanning the 1994-2007 period, report varying antibody prevalence rates at the animal-level that can reach as much as 7% in cattle and, roughly, 10–18% and 10% in sheep and goat, respectively, depending on the region and screening method (Fig. 1). *MAP* infection in slaughtered domestic pigs was also documented^35^. More recently, regional studies with small sampling sizes reported MAP occurrence in wildlife, specifically wild boar, red deer and also carnivores^36–38^ (Fig. 1). Whether *MAP* is self-maintained in these wild populations or arise from animal contact with external sources of infection is yet undetermined. Interaction of these wild species with contaminated faeces from infected ruminants, feeding and scavenging on infected prey, or sympatry with yet unidentified reservoir species, possibly contribute to the dynamic maintenance of *MAP* in the wild, in Portugal.

Control of paratuberculosis can only be achieved through identification of *MAP* host range and transmission paths linking the livestock-wildlife interface to adjust surveillance and ascertain biosecurity measures. As such, the main objectives of this work were to investigate the ecological spread and spill-over of *MAP* into non-paradigmatic hosts, contributing to the disclosure of wild species that potentially may be exposed to this pathogen in mainland Portugal, fine-tuning the proportion of exposed animals and the spatiotemporal distribution of *MAP* in sympatric carnivores. For this purpose, widely distributed animal specimens from the *Mustelidae*, *Herpestidae*, *Viverridae* and *Canidae* families, sampled during a 10-year period across the whole territory of the mainland, were opportunistically surveyed for the presence of this bacterium using a polyphasic diagnostic approach based on microbiological and molecular methods. We also aimed at identifying environmental (climate and orography), bio-ecological (food resources, population and individual factors), and disturbance factors (human and cattle) that correlate with *MAP* infection in carnivores which might help to understand this pathogen’s ecology and serve as predictors for ascertaining populations at risk.

## Methods

### Biological specimen collection and demographic characteristics

The presence of MAP was opportunistically investigated in widely distributed free-ranging animals from four families of the Carnivora order (*n* = 202): Egyptian mongoose (*Herpestes ichneumon*, *Herpestidae* family, *n* = 149), red fox (*Vulpes vulpes*, *Canidae*; *n* = 40), common genet (*Genetta genetta*, *Viverridae*; *n* = 5), stone marten (*Martes foina*, *Mustelidae*; *n* = 4) and European badger (*Meles meles*, *Mustelidae*; *n* = 4), collected between 2005 and 2014 in mainland Portugal (Supplementary Table 1; Supplementary Fig. 1). The surveyed animals were either found dead on the road (n = 28) or hunted during legal hunting actions aiming the control of predator densities (*n* = 174). Corpses were donated for scientific purposes by road maintenance companies, hunters, game reserve owners or hunting associations. No animals were sacrificed for the purposes of this specific study. None of the authors were responsible for the death of any animals nor were any samples used in the study collected by the authors. All applicable institutional and/or national guidelines for the care and use of animals were followed.

Each specimen was assigned to one of four age classes (adults, sub-adults, juveniles and cubs), according to body length and dental growth^39,40^. Animals were geo-referenced upon collection.

### Diversity analyses to assess sampling bias

A diversity analyses to ascertain adequacy of sampling for epidemiological purposes was performed, considering the constraints related to our opportunistic strategy. The relationship between abundance and distribution of samples was established by linear regression; the calculation of diversity indices (Shannon-Wiener, Simpson and Berger-Parker), according to Help and collaborators^41^, and the determination of non-parametric estimators (chao 1 and chao 2) of species richness, according to Gotelli and Cowell^42^, were also performed. Mathematical formula used for these calculations are provided in Supplementary Table 2.

### *Post-mortem* examination

Animal carcasses were frozen at −20 °C (most for up to two months, although a few had been stored for one year) until *post-mortem* examination. The carcasses were thawed and examined. Those macroscopically showing clear signs of putrefaction, or in evident poor preservation condition, were eliminated from the study. A portion of the spleen was removed aseptically to sterile containers, refrozen at −20 °C and later processed for bacteriological culture and nucleic acid extraction as described below. The abdominal cavity of each specimen was opened and the intestines isolated. Solid intestinal content was collected from the rectum of each animal, using a sterile feces collection tube, refrozen at −20 °C, which was subsequently processed for further analysis (Supplementary Table 1).

Fecal (*n* = 111) and/or spleen (*n* = 149) samples were tested by microbiological culture and/or molecular analysis in the scope of a master thesis^43^. No gross lesions suggestive for *MAP* were detected during necropsy, even though histopathological examinations performed in the scope of another study (unpublished data) evidenced autolysis of a proportion of specimens.

All necropsy procedures and subsequently sample collection followed all applicable institutional guidelines and regulations.

### Bacterial culture of feces and spleen

For bacterial culture purposes, 1 g of feces and 2 g of spleen samples removed aseptically from the containers were macerated and homogenized with 20 ml sterile water or sterile saline, respectively. After homogenization, 5 ml were maintained at 4 °C until further processing for DNA extraction, and other 5 ml were decontaminated with 0.9% (v/v) or 0.7% (v/v) of hexadecylpyridinium chloride monohydrate (HPC) (*Sigma-Aldrich*), respectively, following the methodology described in OIE manual guidelines (2008). After decontamination, the sample was centrifuged and the sediment inoculated (100 μL) into slants containing standard cultivation media: Herrold’s egg yolk medium (HEYM) supplemented with mycobactin J and HEYM without mycobactin (*Biogerm*, Portugal). *MAP* positive controls (K10 strain) and physiological saline blanks were seeded in solid media in parallel with each processed batch for culture. After inoculation, the tubes were maintained at 37 °C and observed for bacterial growth every week. The incubation period was at least 6 months. Bacterial culture procedures were carried out in a biosecurity level 2 facility, in accordance with the experimental protocols implemented and approved by INIAV, the national reference laboratory (NRL) for animal health, Portugal.

Microbiological culture in HEYM with and without mycobactin J was attempted for all the fecal samples. Subsequently, based on the positive results from nested real-time IS*900* PCR, nine spleen samples from feces-positive specimens were also processed for culture.

### DNA extraction from fecal and tissue suspensions

DNA extraction directly from 5 mL of feces or spleen suspensions was performed using the commercial system Invisorb^®^ Spin Tissue Mini Kit (*Stratec Molecular*) and High Pure PCR Template Preparation Kit (*Roche*), respectively, following the manufacturer’s instructions. Despite using commercial systems, we optimized nucleic acid extraction by including additional steps for the concentration of samples and the mechanical disruption of cells in order to maximize sensitivity. A bead-beating protocol was used for mechanical disruption, before applying Proteinase K. Zirconium beads were added to sample suspensions in lysis buffer and 6.5 ms^−1^ cycles for 45 sec, repeated twice, were performed in the FastPrep FP120 Bio101 (*Savant Instruments*). Disrupted samples were cooled on ice for 15 minutes, 50 µL of proteinase K was added, and the resulting mixture was incubated overnight at 52 °C. The remaining procedure was performed according to the manufacturer’s instructions. The genomic DNA was eluted with 100 µL of elution buffer and stored at −20 °C until further use. Blanks (negative tissue or saline) were included to discard contamination during extraction. A bovine fecal sample known to be *MAP*-positive by culture was also included in several batches of DNA extraction to confirm that the procedure would adequately extract *MAP* DNA. All steps were carried out in a laminar flow cabinet and precautions were taken during all stages to avoid nucleic acid carry-over. Obviously, only experiments wherein negative and positive controls performed correctly were considered.

### Nested real-time PCR for the detection of IS*900* insertion sequence

The detection of *MAP* in the genomic DNA extracted from biological samples was performed by a novel nested real-time PCR approach targeting IS*900*, developed at INIAV (LNR) in the scope a PhD thesis^44^. The genomes of *MAP* strains contain 15–20 copies of IS*900*^45^ making this a good target for molecular diagnosis purposes. This new in-house IS*900*-PCR includes modifications of a previously validated real-time PCR system optimized for human samples^46^ in order to increase diagnostic sensitivity in animal samples^44,47^. These newly designed primers (forward: 5′-TGATCTGGACAATGACGGTTACGGA-3′; reverse: 5′-GGCGTTGAGGTCGATCGCCCACGTGAC-3′) are located within IS*900* but are external to the amplicon generated by the methodology described by Sidoti and co-workers^46^. They target a fragment of 224 bp, located between positions 204 e 427 of IS*900* sequence. The second amplification step, amplifying an internal IS*900* sequence, maintained the primers and probe described by Sidoti *et al*.^46^. The specificity of both primer pairs and hydrolysis probe sequences was assessed against the NCBI nucleotide sequence database. The *in silico* analysis showed 100% identity between the nucleotide sequences of the primers and probe with the homologous sequences in *Mycobacterium avium* subsp. *paratuberculosis*, confirming specificity of this molecular procedure to detect *MAP*.

The PCR reaction was carried out in a final volume of 25 μl containing 1x NZYTaq Colourless Master mix (*NZYTech*), 0.5 μM of each primer, DNase-free water and 5 μl of extracted DNA template. The first amplification reaction was performed in C1000 Thermal Cycler (*Bio-Rad*) at 55 °C (annealing temperature) and the amplified products were stored at 4 °C, until electrophoretic analyses in a 2% agarose gel prepared in TBE (Tris-Borate-EDTA) 1x, or directly used in the second real-time PCR step. The second real-time amplifies a 66 bp sequence within the first amplicon using primers, probe and experimental conditions of Sidoti and collaborators^46^. This PCR was performed using as template 5 μL of the amplification product from the first PCR. Real-time PCR reactions were performed in CFX96 Real-Time Thermal Cycler (*Bio-Rad*) and the amplified products were stored at 4 °C until electrophoretic analyses in a 2% agarose gel. Fluorescence measurements were recorded after each annealing step (at 60 °C) and data was analyzed with the software Bio-Rad CFX Manager 3.0 (*Bio-Rad*) and in relation to standard calibration curves prepared as described below. For all calculations, the baseline and the fluorescence threshold were set automatically.

In all PCR batches, in addition to samples, positive controls (DNA extracted from *MAP* ATCC 19698^T^ and DNA from a positive bovine fecal sample) and a no-template control (water) were included. Since a nested approach was used, multiple negative controls (uninfected bovine feces extracted as described up above and no-template) interleaved with samples were included for PCR to discard cross-contamination and pseudopositives. No-template and positive controls were included in each single assay (including DNA extraction, molecular detection, and also culture steps). All samples were tested by PCR (standard and real-time) at least three times and considered positive or negative if yielding coherent results in at least two independent amplification experiments. Only experiments wherein negative and positive controls performed correctly were considered. PCR steps (reaction mix preparation and template addition) were carried out in different laminar flow cabinets located in separate rooms, according to routine laboratorial practices at INIAV IP. Precautions were taken during all stages to avoid nucleic acid carry-over. For quality control purposes, six positive and 16 negative samples were re-extracted and re-tested by PCR. Result agreement was 100%. In several occasions, the amplified products from both PCR reactions were analyzed in 2% agarose gels prepared in TBE 1x and amplicons checked for the expected size. This procedure was always employed whenever the threshold cycles (Cq) of the real-time PCR analyses crossed 35, to confirm amplification of fragments with expected size.

An animal was classified as *MAP*-positive if the bacterium was isolated from at least one biological matrix in selective media and/or if *MAP* IS*900* was detected by means of PCR at least twice (out of three independent tests).

### Performance of the IS*900* nested real-time PCR

Spiked faecal samples with ten-fold dilutions of a suspension of *MAP* cells were used to assess detection limits (LOD) of the nested approach^44^. Faecal samples were pooled from cattle that tested negative for paratuberculosis by traditional culture, immunological and PCR tests, and showed no histopathology consistent with paratuberculosis following slaughter. Six grams of pooled faeces was spiked with ten-fold dilutions of a suspension of *MAP* cells, either with the *MAP* K10 (Type C) or *MAP* 235G (Type S) strains, in a range of 10^4^ to 10^1^ cells per gram of faeces, tested in triplicate. An additional 6 g of faeces without *MAP* cells was used as a negative control. The number of *MAP* cells was estimated by microscope count. The tubes were mixed well and stored at −20 °C until required. The genomic DNA of the faecal spiked samples was extracted and tested by nested real-time PCR approach targeting IS*900* with the same procedures as the faecal samples^44^.

The performance of this novel PCR assay was tested^44^ using a set of 75 ruminant faecal samples, showing a diagnostic sensitivity of 96.6% and a kappa coefficient of 0.60 (moderate strength of agreement) when compared with culture gold standard^44^. The sensitivity of this nested approach was shown to be superior to that of the single-step real-time PCR^46^ when this was applied as a single approach to the same samples (96.6% versus 74.6%, respectively)^44^. For *MAP* Type C, positive amplification results were detected at an infection rate of 10^1^ cells per gram of faeces, while for Type S the detection limit was 10^2^ cells per gram^44,47^, thus showing that this procedure is superior in its ability to detect *MAP*.

Nineteen reference, clinical and environmental strains of *Mycobacterium avium* complex (MAC) (M. *avium* subsp. *hominissuis*, *M. avium* subsp. *avium*, M. *avium* subsp. *paratuberculosis*, *M. scrofulaceum*), non-MAC mycobacteria (*M. bovis* BCG, *M. tuberculosis*) and non-mycobacterial (gram positive and gram-negative) clinical species maintained at INIAV were used^44^. Only *MAP* strains yielded amplification of IS*900* with the amplification system herein described (100% specificity and sensitivity), confirming that this novel PCR methodology is fully specific to detect *MAP*.

The analytical sensitivity, specificity and kappa coefficient were computed using the previously mentioned panel of strains and samples and the publicly available clinical research calculators on VassarStats^48^ website (http://vassarstats.net).

The details of these methodologies and the validation work are fully described elsewhere^44^.

### Sequence analyses of IS*900* nested real-time PCR amplicons

Three 224 bp amplicons from red fox, mongoose and genet, generated from the first PCR round with the newly established IS900 primers were excised from the agarose gel, purified using QIAquick gel extraction kit (*Qiagen*) and commercially sequenced (*GATC Biotech* and Eurofins). The obtained sequences were aligned using *Basic Local Alignment Search Tool* (BLASTn) in order to find regions of local similarity with sequences from the database. Eleven IS*900* sequences from 11 *MAP* strains genomes (Accession numbers AJ250023.1, AJ250018.1, AJ250016.1, AJ250017.1, AJ250015.1, AJ250021.1, AJ250022.1, S74401.1, AF416985.1, X16293.1 and HM015765.1) and two IS*900*-like sequences (from *Mycobacterium* sp. 2333, accession number AF455252.1; *Mycobacterium porcinum*, accession number EU126150.1) were retrieved from NCBI nucleotide database and aligned with the amplicon sequences obtained in this work using BioEdit Sequence Alignment Editor v7.0.5.3.

### Real-time PCR for the detection of *MAP* F57 sequence

Confirmatory testing of IS*900* positive samples was attempted by a specific real-time PCR assay for the single-copy *MAP* F57 sequence, following the procedure, primers and probe described by Sidoti and collaborators^46^. Real-time PCR assays aiming the amplification of a 80 bp fragment were performed using QuantiFast Pathogen PCR + IC Kit (*Qiagen*). This kit includes an Internal Amplification Control (IAC), as well as specific reagents and primers/probes required for its amplification. The optimized setup with a final 25 μl volume per reaction, contained 1x QuantiFast Pathogen Master Mix, 1x IAC assay, 1x Internal control DNA, 0.4 μM of each primer, 0.2 μM of probe, DNase-free water and 5 μl of DNA template. All PCR reactions were also performed in CFX96 Real-Time Thermal Cycler (Bio-Rad) according to the following cycling conditions: 95 °C for 10 min, followed by 45 two-step cycles of 95 °C for 15 s and 60 °C for 1 min, with data acquisition at this step. The software Bio-Rad CFX Manager 3.0 (Bio-Rad) was used for data analysis. The baseline and the fluorescence threshold were set automatically. In all PCR batches, in addition to samples, positive control (DNA extracted from MAP ATCC 19698^T^) and a no-template control (water) were included. The products of amplification reactions were stored at 4 °C until electrophoretic analyses in a 2% agarose gel.

### Statistical analyses of the mongoose’s results

Chi-square test and Mann-Whitney test (α = 0.05) were performed whenever appropriate in IBM SPSS Statistics 22.0; and graphics were produced in Excel and GraphPad Prism 6.0. Significant statistical differences were established at p < 0.05. The confidence intervals (95% CI) were calculated online on the website: VassarStats: Website for Statistical Computation^48^. Based on the known ecology of mongoose^40,49–52^ and *MAP* occurrence in ruminants, we tested the influence of variables that could directly or indirectly influence *MAP* infection (using IS*900* detection as proxy) in mongoose populations, the only carnivore species with enough samples to allow a modeling procedure. Thus, we considered five categories of variables, representing different working hypotheses: H1) productivity/food resources availability (Normalized Difference Vegetation Index-NDVI, wild rabbit and red-legged partridge abundances); H2) disturbance (human population density and road network density); H3) life-history traits [(gender, age and morphometry (e.g. body size), mongoose abundance]; H4) Climate and orography [season, average annual temperature, annual rainfall, and annual temperature range- data were calculated by interpolations of observed data, representing average values for the period 1960–1990^53^; altimetry, river network density]; and H5) Cattle (density of cattle farms). Each variable was represented by mean values of the 2 × 2 km grid cell, considering the home range of the Egyptian mongoose^54^. Climate data comprised variables gathered from BioClim (2015)^55^ at 30 arc-second resolution^53^. The Normalized Difference Vegetation Index (NDVI) was used as a proxy of primary productivity^56^. NDVI values for each record was calculated from satellite images supplied by Moderate Resolution Imaging Spectroradiometer^57^ at a spatial resolution of 250 m. The elevation data (altimetry) was computed using the ASTER Global Digital Elevation Model platform^58^. Hydrographic data (river network) was gathered from the Sistema Nacional de Informação de Recursos Hídricos^59^. The human population density was derived from the European Commission (2015) and road network from IGP (2015)^60^. Latitude and longitude were used as a measure of geographic position of the collected samples. Abundances of Egyptian mongoose, European rabbit (*Oryctolagus cuniculus*) and red-legged partridge (*Alectoris rufa*) were estimated based on annual hunting yields published by the National Authority for Nature Conservation (Instituto da Conservação da Natureza e Florestas/ICNF). Cattle breeding farms density per municipality, i.e. farms/km^2^, (provided by the National Veterinary Authority, Direção Geral de Alimentação e Veterinária/DGAV) was also included as variable given the occurrence of *MAP* in livestock and its potential spill-over to wildlife.

Body size was estimated as the first component of a principal component analysis (PCA) performed to enable a single estimate based on the covariance matrix of various measures. Calculations included combining weight and six biometric measurements as described by Bandeira *et al*.^40^ [snout-tail length (terminal hairs not included), right hind leg length, right hind foot length, shoulder height, neck perimeter and head width)] of each mongoose specimen. Variables were tested for normality with Kolmogorov-Smirnov Test (with correction of Lilliefors for the significance level)^61^. The categorical co-variates “Gender” and “Season” were composed by two and four categories, respectively. For “Gender” the defined reference category was “Female” and for “Season” we selected “Winter”.

Regarding the modelling procedure, first we tested for data spatial autocorrelation, to account for the degree of geographical dependency of data, using the Moran’s I index with the R’s “ape” package^62^. Multicolinearity was tested by estimating the Spearmans correlation rank (r)^61^ and the statistical significance between variables included in two groups of variables: (1) wildlife and cattle abundance (Egyptian mongoose, wild rabbit and partridge abundances and cattle farms density); and (2) climatic and disturbance factors (human density, road network, river network, altimetry, average annual temperature, annual temperature range, annual rainfall and NDVI). When the correlation coefficients (r) between two variables were equal or superior to 0.7, we excluded those that presented a lower correlation with the dependent variable (i.e. *MAP* IS*900* detection)^63^.

All the remaining continuous candidate variables were standardized prior to the model building procedure to facilitate results interpretation and modelling procedures. Due to the low detection rate of IS*900*, we used a Logistic Regression in Rare Events data approach (LRRE) to test which variables might be driving/constraining detection in Egyptian mongooses. This approach is adequate to study binary events in situations where non-detections are hundreds or thousands of times more frequent than detections^64^, as it controls for the underestimation of occurrence that might take place when using simple logistic regression approaches in such situations^64^. Modelling procedures were implemented using the “logistf” package^65^ in R 3.1.2.

We implemented a two-folded analytical procedure. First, we used only the variables associated to each working hypothesis (see above), and built several model for each hypothesis, corresponding to all combination of variables associated to the hypothesis. Then we used an Information Criteria approach (Akaike Information Criteria, corrected for small samples – AICc)^66^ to select the best models for each hypothesis. All produced models were ranked according with their ΔAICc (corresponding to the difference between the model’s AICc and the smallest AICc value). Those models with a ΔAICc < 2 were selected as best models^66^.

In a second stage, we compared the best model(s) for each hypothesis, using the AICc approach, to test what hypothesis was more supported by our data. As more than one model fulfilled the best model criterion (i.e. ΔAICc < 2), we implemented a model averaging procedure to estimate variables coefficients. Variables with coefficients 95% confidence intervals that did not include zero, and with a significant relation with the dependent variable, were those considered in the discussion (as only for those we could assess if their influence in *MAP* detection was significantly positive or negative). Odds-ratio for each variable included in the best model(s) were also estimated. Model selection procedures were implemented in R package “MuMIn”^67^. Best model validation was tested by calculation the Receiver Operating Characteristic (ROC) Curve, and the Area Under the Curve (AUC)^68^, using the R package “pROC”^69^.

## Results

### Demography and diversity analyses of the samples under study

Two-hundred and two carnivores belonging to five carnivore species (Egyptian mongoose, red fox, common genet, stone marten, and European badger) were surveyed in this study. Egyptian mongoose and red fox were the most represented species (73.8% and 19.8%, respectively). The majority of surveyed individuals were adult (*n* = 168; 83%). Regarding geographical distribution, the districts of mainland Portugal (first level administrative divisions; *n* = 18) were considered the sampling unit. Five districts (Braga, Porto, Bragança, Viana do Castelo and Setúbal) were not represented in our sampling effort. Most individuals originated from Beja (*n* = 68), while Vila Real, in the inner North, was the district with more animal species analysed (four species) (Fig. 1 and Supplementary Fig. 1).

A diversity analysis was performed to assess if our eventually biased sampling would enable proper inferences on *MAP* occurrence and the underlying epidemiological situation. A positive relationship between the abundance of animals from a particular species and their distribution across districts was registered by linear regression (r^2^ = 0,6152), meaning that the two species from which more individuals were sampled (mongooses and foxes) are indeed the species that are distributed in a higher number of geographic locations. The diversity indices Shannon-Wiener, Simpson and Berger-Parker represent a coherent system for diversity estimates and allow the appraisal of the balance between the number of individuals and the number of species under analysis in a given community (district)^70^. Since only one animal species was sampled from the districts of Leiria and Faro, this analysis was not carried out for those regions. Regarding the estimated diversity indices, the higher values were attained for the districts of Vila Real and Aveiro, which thus can be considered the most diverse and balanced sampled communities (Supplementary Table 3); in contrast, Beja and Lisboa were the most unbalanced. The same diversity indices were re-calculated for clustered geographical areas, the statistical terrestrial units (NUTS). The North region, which includes Vila Real, is, according to this criteria, the most balanced community amongst those of our study (Supplementary Table 3).

Non-parametric estimators of species richness chao 1, which examines species abundance data, and chao 2, that considers species incidence data, were also calculated, evidencing completeness of the sampling method of 100% and 91%, respectively (Supplementary Table 3).

### Detection of *MAP*-positive animals using IS*900* as proxy

*MAP* bacteria were not isolated from −20 °C preserved tissues in none of the selective media used, but *MAP* IS*900* was detected by nested real-time PCR in specimens from four out of the five surveyed species, the exception being the badger (Table 1, Supplementary Table 4). The samples from which amplification of IS*900* was accomplished (*n* = 15) were also tested for F57 by real-time PCR. Amplification was only obtained for the positive control (supplementary Figure). The internal amplification control (IAC) performed correctly for all samples (30 < Ct < 33). Eletrophoretic analyses of the PCR reaction products evidenced an amplicon corresponding to the IAC in each sample but no F57 amplicon (with the exception of the positive control). *MAP’* IS*900* identity of three IS*900* amplicons generated by the first step of the nested approach was confirmed by sequencing using first amplification primers. Alignment with eleven IS*900* sequences from 11 *MAP* strains genomes deposited in Genbank and two IS900-like sequences from *Mycobacterium* sp. and *Mycobacterium porcinum* evidenced 100% similarity of the red fox, mongoose and common genet IS*900* fragments with genuine IS*900* from *MAP*, in parallel with the lack of the point mutations that IS*900*-like exhibit, allowing distinction of IS*900* from IS*900*-like (Supplementary Fig. 2) and thereby confirming the specificity of the carnivore amplicons as IS*900* and the ability of this novel PCR to detect *MAP* using as a proxy IS*900*.

**Table 1:** Characteristics of MAP DNA-positive animals.

Species	Gender	Age class	Year	District	Cause of death	DNA-positive matrix
Red fox (*Vulpes vulpes*) *n* = 40	F	Adult	2010	Vila Real	H	Feces
	M	Adult	2010	Portalegre	H	Feces
	F	Adult	NA	Évora	H	Feces^a^
	F	Adult	2011	Aveiro	RK	Spleen
Egyptian mongoose (*Herpestes ichneumon*) *n* = 149	M	Adult	2011	Lisboa	H	Feces
	M	Adult	2011	Faro	H	Spleen
	M	Adult	2011	Beja	H	Spleen
	F	Adult	2011	Beja	H	Spleen
	M	Adult	2011	Viseu	H	Spleen
	F	Adult	2011	Beja	H	Spleen
	F	Adult	2011	Beja	H	Spleen
	F	Adult	2011	Beja	H	Spleen
	M	Adult	2011	Faro	RK	Spleen
Common genet (*Genetta genetta*) *n* = 5	F	Juvenile	2009	Viseu	RK	Feces
Stone marten (*Martes foina*) *n* = 4	M	Adult	2005	Aveiro	RK	Feces

Legend: F-female, M-male; cause of death: H-hunted, RK-road-killed; ^a^Spleen sample was also tested; NA - not available.

According to established positivity criterion (see methods), 15 out of the 202 animals tested were considered *MAP* IS*900*-positive [7.43% (95% CI: 4.55–11.89%]: nine Egyptian mongoose, four red fox, one stone marten and one common genet collected in 2005 (*n* = 1), 2009 (*n* = 1), 2010 (*n* = 2) and 2011 (*n* = 10). There were DNA positive animals among the road-killed (*n* = 4) as well as the hunted (*n* = 11). Most DNA-positive specimens were adult (*n* = 14; 93%) (Table 1, Supplementary Table 4).

In the most represented species, the percentage of IS*900*-positive animals was 6% (95% CI: 3.2–11%) for Egyptian mongoose and 10% (95% CI: 4.0–23%) for red fox.

*MAP* IS*900* was detected in both feces and spleen samples (5.4% and 6% of total samples, respectively). DNA detection in the spleen of positive specimens was confirmed for a subset of mongooses and foxes (n = 9, 60%). The presence of DNA in feces was confirmed in all four positive species (n = 6, 40% of positive specimens), including 75% of DNA-positive foxes and 11% of DNA-positive mongooses. Statistical analysis by the Chi-square test between feces and spleen samples evidenced no significant difference in the probability of detecting *MAP* IS*900*in any of the biological matrices tested (p > 0.05).

The distribution of Cq values among fecal and spleen samples ranged from 7.74 to 37.41 and from 10.28 to 44.08, respectively. Amplification reactions yielding Cq above 40 were considered a positive result if amplification of the 66 bp fragment was confirmed by electrophoresis. The mean Cq values were 23.0 and 27.3, respectively (Supplementary Fig. 3). The mean Cq values of the two matrix types under analysis were compared by the Mann-Whitney test and no significant differences were obtained (i.e. p > 0.05). The mean Cq values obtained for biological samples from the most represented hosts (red fox and Egyptian mongoose) were also compared by the Mann-Whitney test and, again, no significant differences were found (i.e. p > 0.05; Supplementary Fig. 3).

Regarding the geographic location, IS*900*-positive animals were detected in eight out of the 13 (61.5%) districts analyzed (Fig. 1 and Supplementary Table 4). None of the animals sampled from the central region of the mainland (Santarém, Coimbra, Guarda, Castelo Branco and Leiria districts) tested positive, while Beja, from where most animals were sampled, was the district with more IS*900*-positive specimens (*n* = 5; 7.35%). Faro and Aveiro presented the highest proportion of DNA-positive animals (40.0% and 66.7%, respectively) (Fig. 1 and Supplementary Table 4).

### Unraveling significant predictors for *MAP* using IS*900* detection in mongooses as proxy

The influence of productivity/food resources availability, disturbance, life-history traits, climate and orography, and density of cattle farms, which potentially could influence *MAP* detection using IS*900* as proxy was investigated through modeling strategies on PCR positive and negative mongooses, whose representative sampling (*n* = 149), in comparison with the remaining surveyed species, would enable robust modeling analyses. Five specimens were excluded as gender information was not available.

The components retained from principal component analysis (PCA) of body size (based on weight, snout-tail length, neck perimeter) had loadings higher than 0.70. PCA of body size for each specimen was explained by a proportion of variance of 85.6%, with an eigenvalue of 2.568. No significant spatial autocorrelation of the collected samples was detected (Moran’ I = −0.006; P = 0.956), indicating a negligible influence of spatial dimension on our data structure. Multicollinearity testing indicated that several variables among those selected as potential predictors were highly correlated: wild rabbit and red partridge abundance: r = 0.69, P < 0.001; human density and road network: r = 0.84, P < 0.001; average annual temperature and annual rainfall: r = 0.92, P < 0.001; altimetry and average annual temperature: r = 0.79, P < 0.001; altimetry and annual rainfall: r = 0.91, P < 0.05. Thus, partridge abundance, human density and average annual temperature were excluded from the analyses based on lower rho correlation estimates from Spearmans correlation rank test with the dependent variable. Thus, we tested all combinations of the following candidate predictors: H1 - NDVI and Abundance of wild rabbits; H2 - Road network; H3 - Gender, Age, Body size and abundance of Egyptian mongooses; H4 - Season, Annual temperature range, Annual rainfall, Altimetry, and River network; H5 - density of cattle farms. Although several models were identified as the best models for each working hypothesis (see Table 2), those related with the fourth hypothesis, i.e. Climate and orography hypothesis, were the ones that reached lower AICc values and a ΔAIC < 2), thus representing the most parsimonious and explanatory hypothesis to describe the probability of IS*900* detection in mongoose (Table 2). Those models were composed by a combination of four variables (Tables 2 and 3). Model averaging procedures of the two best models indicated that annual temperature range, annual rainfall and altimetry as the variables with most significant relative importance (with p < 0.05) and with coefficients 95% confidence intervals that did not cross zero, enabling to determine the type of effect (Table 3). Annual rainfall and annual temperature range exerted a negative influence on MAP infection probability in mongooses (with an odds-ratio of 0.034 and 0.146, respectively), while altimetry had a positive effect (with an odds-ratio of 21.575) (Table 3). The AUC value derived from the ROC curve reached 0.855, revealing good accuracy of the average model to predict *MAP* infection in carnivores^71^.

**Table 2:** Best models for each of the working hypothesis selected according to the model’s AICc and ΔAICc. The hypotheses and models with the higher support (and lower AICc values) are presented in bold.

Models	df	AICc	ΔAICc	AICc weight
H1 - productivity/food resources availability hypothesis				
NDVI + Rabbit_abundance	3	65.20	0.00	0.419
NDVI	2	65.34	0.14	0.392
Rabbit_abundance	2	66.79	1.59	0.189
H2 - Disturbance hypothesis				
Road_network	2	67.04	0.00	1.000
H3 - Life-history traits hypothesis				
Body_size + Age	3	61.67	0.00	0.162
Body_size + Age + Mongoose_dens	4	61.67	0.00	0.162
Age + Mongoose_dens	3	61.98	0.31	0.138
Age	2	62.03	0.36	0.135
Gender + Body_size + Age	4	62.97	1.30	0.085
Gender + Body_size + Age + Mongoose_dens	5	63.00	1.33	0.083
Gender + Age + Mongoose_dens	4	63.24	1.57	0.074
Gender + Age	3	63.26	1.59	0.073
H4 - Climate and orography hypothesis				
**Altimetry + Annual_rainfall + Annual_temp_range + River_network**	**5**	**55.87**	**0.00**	**0.542**
**Altimetry + Annual_rainfall + Annual_temp_range**	**4**	**57.61**	**1.74**	**0.227**
H5 – Cattle farm hypothesis				
Dens_cattle_farm	2	66.84	0.00	1.000

df - degrees of freedom. AICc - Akaike’s information criterion. ΔAICc - difference to the lowest AICc value; AICc weight - Akaike weights. Rabbit_abundance - Abundance of wild rabbits; NDVI - Normalized Difference Vegetation Index; Road_network - road network density; Body_size – First component of the Principal component analysis (PCA) of body size (based on weight, snout-tail length, neck perimeter); Mongoose_dens – Egyptian mongoose abundance; Annual_temp_range - annual temperature range.

**Table 3:** Averaged coefficients and relative importance of the variables included in the best models considered explanatory for *MAP* detection in Egyptian mongoose in Portugal (H4 - Climate and orography hypothesis). Significance was established at P < 0.05. Variables whose CI95% did not include 0 are indicated in bold.

	Estimate	Std. Error	z-value	P	95%CI	Odds-ratio	Relative importance
(Intercept)	−3.722	0.796	4.678	<0.001	−5.282/−2.163		
**Altimetry**	**3.072**	**1.327**	**2.315**	**0.021**	**0.471/5.673**	**21.575**	**1**
**Annual_rainfall**	**−3.380**	**1.585**	**2.133**	**0.033**	**−6.486/−0.274**	**0.034**	**1**
**Annual_temp_range**	**−1.925**	**0.742**	**2.593**	**0.010**	**−3.380/−0.470**	**0.146**	**1**
River_network	0.521	0.543	0.960	0.337	−0.253/1.732	2.095	0.7

z-value −; P − p-value; 95% CI – 95% Confidence Interval; Annual_temp_range - annual temperature range.

## Discussion

The high apparent prevalence rates that have been found in livestock populations over the years, together with two recent reports of regional distribution in wild boar and red deer^36,37^, suggest that *MAP* occurrence in mainland Portugal is enzootic (Fig. 1). Thirteen out of 18 administrative regions from mainland Portugal were represented in our sampling effort. Despite the constraints related to an opportunistic strategy, species diversity, abundance and richness estimates, calculated with different algorithms/indicators at the district and NUTS levels, suggest that the mesocarnivore communities sampled here were, in general, balanced, both in composition and structure, and that our strategy is appropriate to appraise *MAP* occurrence and ecological interactions. Although sample sizes were intrinsically limited for low-density *Mustelidae* and *Viverridae* species, we show that important terrestrial species of the Mediterranean carnivore guild, such as stone marten and genets, may be exposed to *MAP*, while we provide the first report of *MAP* IS*900* detection in common genet. Our survey is also the first to report IS*900* in feces from wildlife in Portugal as this matrix was not tested in any of the previous surveys, although fecal shedding was reported in bovines from the North of Portugal^72^.

We found epidemiologically relevant proportions of *MAP* IS*900*-positive animals among extensively sampled wild carnivores from the *Canidae* and *Herpestidae* families, particularly red fox (10%) and Egyptian mongoose (6%). Detection of positive wild carnivores from Northeastern Portugal down to Alentejo (Beja) and further south, in the Algarve, represents important new data, since all the information available so far on *MAP* incidence was limited to free-ranging animals from the Centre and Northeast. This is particularly important in terms of conservation because Guadiana Valley region (in Beja district) is a conservation priority area for some Portuguese menaced predators, such as the Iberian lynx (*Lynx pardinus*), Spanish Imperial eagle (*Aquila adalberti*), and the European wildcat (*Felis silvestris*)^73–75^, which might be susceptible to *MAP*. Iberian lagomorphs such as wild rabbit (*Oryctolagus cuniculus*) and Iberian hare (*Lepus granatensis*) also occur in this region. Potential exposure to *MAP* is also of relevance since these two species are already very fragilized in mainland Portugal due to rabbit hemorrhagic disease (rabbit) and myxomatosis (rabbit and hare) epizootics in recent years.

A previous report described *MAP* isolation or *MAP* DNA detection in 27% of 74 surveyed wild carnivores from the central region of mainland Portugal^38^. Although our study design included almost three times more animals than those surveyed by Matos and collaborators (2014)^38^, the mean proportion of exposition to *MAP* using IS*900* as proxy we estimated is much lower, around 7.4% (95% CI: 4.5–11.9), with considerably fewer positive mongooses [6% (95% CI: 3.2–11%), relative to the 46.7% reported by Matos *et al*. (2014)]^38^. Regarding red fox, our estimates are also underneath those of Matos *et al*. (2014) [10% (95% CI: 4.0–23%) versus 14% determined by Matos *et al*. (2014)]^38^. Also in contrast, none of the animals that we sampled from the central region of the country tested positive for *MAP* IS*900*. Differences in estimated proportions by both studies might be related to several factors, namely lower number of matrices tested per specimen in the current work, prolonged sample storage diminishing detection probability and the fact that our sampling array spans a longer time period (ten versus four years) and a wider geographical area. Furthermore, it is possible that sites tested by Matos *et al*.^38^ might be located near livestock herds with higher *MAP* prevalence than herds near our sampling sites, resulting in spillover to wildlife. However, we lack data to test this idea. Furthermore, since *MAP* bacterial isolation is hampered in the present work, we cannot discard the hypothesis of having detected a non-culturable (myco)bacteria present in small amounts in mongoose and in other carnivore species that has an IS*900* sequence undistinguishable from the one present in *MAP*.

Intersecting data from the studies published so far in Portugal suggests that *MAP* may have a wider natural host range than previously supposed and that enzootic occurrence in wildlife may be a recent phenomenon, occurring in parallel in different geographical areas and possibly related with different infection sources. However, we also cannot discard the hypothesis of *MAP* expansion from “traditional” hotspots, since we detected IS*900*-positive animals both in areas formerly recognized as positive but also in novel ranges.

*MAP* occurrence among sympatric wild populations is expected to be influenced by ecological interactions dependent on behavior, social and spatial structure, and also by physiological characteristics of the hosts. The carnivore species that we explore in this study as susceptible are considered habitat and trophic generalist^76^. The surveyed species also explore the environment in different ways and may, therefore, interact with domestic and wild species differently. While common genets and stone martens are arboreal and solitary, with the first predating more on rodents, foxes and mongooses are cursorial predators that use the landscape mainly in two dimensions and, thus, have higher opportunities to contact with domestic animals in rural settings. In spite of being considered as a solitary animal, the observation of mongoose family groups is frequent^77^, indicating a certain tendency for sociability. Besides social behavior, some of these predators, like genets and mongooses, use a promiscuous scent marking behavior, characterized by defecation of several individuals in latrines^78^. This behavior may increase the chances for intra- and inter-specific infection with *MAP* or other mycobacteria due to intermittent shedding in feces and contamination of the environment, namely water and pasture, thus perpetuating the infection cycle. Furthermore, fox and mongoose’ diets include small vertebrates, such as free-ranging wild rabbit in which *MAP* was previously reported, both in Portugal^79^ and Scotland^15,16^. Therefore, in addition to contamination via social interaction and/or the environment, predator infection from ingestion of contaminated prey may also be a plausible infection source.

Besides the effects of host ecological features mentioned above, numerous environmental (e.g. climate), other host biological characteristics (e.g. age, level and source of exposure), and bacterial factors (e.g. infective dose and strain genotype), are hypothesized to affect the natural resilience and burden of *MAP* outside the host, amplifying (or decreasing) the infection risk and routes of transmission. Although based on a single species models, our results clearly suggest that probability of *MAP* IS*900* detection in mongoose was determined by region’s altitude, climate stability and dryness.

The likelihood of mongoose exposure to *MAP* or to a non-culturable bacterium carrying IS*900* was positively associated with higher altitude, narrower annual temperature range (i.e. climate stability) and lower annual rainfall, an overlapping combination that is mostly encountered in the North of Portugal, with the exception of the north western region, where mongooses are currently absent^52^. In this region, 90% of the land is above 400 m, and the highest areas of Portugal are located. The dominant northern lithology in the upper inland is granitoids and a flysh-type series of schists and graywackes^80^, wherein pH is more acidic and topsoil iron, zinc and nickel, are more abundant^81^. Also in this northern region, the temperature range and solar irradiation are less pronounced, rainfall is intermediate, and hydrographic network is denser. Although we did not test for *MAP* environmental presence in these regions, we speculate that the combination of these environmental variables may favour *MAP* survival off-host. Some experimental studies have shown that *MAP* survival correlates with temperature and pH, with the bacterium being moderately susceptible to long-term desiccation, exposure to sunlight, and sensitive to soils with an alkaline pH or low iron content^82^. *MAP* survival for long periods of time in other extreme conditions such as alkaline soils is, on the other hand, underreported. In contrast with the south, the north is also characterized by higher human density and transformation by human action, more fragmented habitats and lower densities of prey target species. Although the number of livestock farms is also higher in the north, we did not detect a significant effect of this factor, which may indicate that this is probably not an important *MAP* infection route for mongoose. Our knowledge about *MAP* distribution and genotypes, the guild of susceptible species across regions, and prevalence rates remains limited owing to several factors, including *MAP* isolation and genotyping difficulties that continue to challenge microbiologists. The results assembled in this work were partially restricted by the lack of isolation of *MAP* and the possibility of DNA extraction from pure cultures, which would have been essential to definitely confirm IS*900*-positive animals as being *MAP*-infected and to complete downstream application of molecular typing techniques. Bacterial isolation achievement would enable the identification of infection sources and of transmission directionality. We thus cannot discard the possibility of having detected a non-culturable bacterium present in small amounts in mongoose and in other carnivore species that has an IS*900* sequence undistinguishable from the one present in MAP. Adding to the extraordinary challenge of culturing *MAP* and its intrinsic extremely slow growth rate, the necessity of aggressive chemical decontamination of samples to eliminate rapidly growing microorganisms often impair bacilli growth up to detectable levels^83^. On the other hand, opportunistic testing of hunter-harvested animal specimens habitually implies using autolysed samples or samples poorly preserved by donors in domestic freezers (<−20 °C), further complicating the recovery of injured bacteria. Indeed, the conditions related to the management of samples (e.g. long term storage, freeze–thawing) and sub-optimal preservation most probably hampered culture success in this work. *MAP* does not survive well over-time at −20 °C. To retain viable bacteria for long-term storage, samples would have needed to be stored at −80 °C. The absence of microbiological isolation in selective media has often been reported by others^47,83–85^ and, while expected in opportunistic studies, it has been the driver underlying several reports informing on more sensitive methods. For diagnosis purposes, we were able to overcome these limitations by using a more sensitive technique, such as nested real-time PCR approach directed towards IS*900*. This procedure has been successfully used to detect *MAP* DNA in feces^44^ and in milk and bulk milk samples^47^. The improvement and validation of this molecular detection system was also performed together with enhancement of the nucleic acid extraction method. Additional steps for the concentration of faecal samples and the mechanical (bead beating) disruption of the cells were included in order to maximize sensitivity.

The IS*900* is defined as a 1,451 bp multicopy element inserted into 15 to 20 conserved *loci* in the *MAP* genome^45^ depending on the strain and has been the marker of choice for most molecular assays. However, as IS*900*-like sequences have been demonstrated in other unrelated *Mycobacterium* species, we conducted F57 PCR^46^ on positive samples from the first step of the nested approach and also sequenced the amplicons from three IS*900*-positive samples to further confirm specificity of *MAP* DNA amplification. Although no F57 amplicon was obtained in carnivore samples, specificity of IS*900* sequences amplified from mongoose and red fox was confirmed by nucleotide sequence alignments of amplicons displaying 100% similarity with genuine IS*900* from 11 *MAP* genomes. Our samples also lacked the point mutations that distinguish IS*900 and* IS900*-like*, thereby confirming the identity of the amplicons as IS*900* and the ability of this novel PCR to specifically detect *MAP* IS*900*.

Although F57 is highly specific for *MAP* detection, it is present only in one copy, and so analytical methodologies based on F57 amplification have reduced lower sensitivity when compared to qPCR for IS*900*. The odds to detect it in poorly preserved samples or in samples with low quantities of DNA are thus lower. The specificity of the external and internal primers and probe used in this survey for IS*900* DNA detection in a nested methodology was originally assessed by homology searches in the NCBI nucleotide sequence database. *In silico* analysis showed 100% identity with the homologous sequences in *MAP* genomes. In parallel with sequencing results, specificity concerns were also ruled out by the performance parameters we obtained when validating the method with a panel of mycobacterial and non-mycobacterial species, thus confirming specificity and accuracy of selected primers to avoid cross-reactive elements, such as IS*900*-*like*.

While the mean Cq values of the nested approach were 23.0 and 27.3 in feces and spleen of positive carnivores, respectively, lower values (but not statistical significant) possibly reflect a higher *MAP* burden in the sample, although better DNA extraction efficiency or better sample preservation may also jointly contribute to lower quantification cycles. Under these low Cq values and according to current understanding of the pathogenesis of the disease, pathology and culture would be expected to be positive. The proportion of detection in parenchymal organs was also anticipated to be lower relative to intestine/feces since systemic infection is considered a late stage event, at least in ruminants. When systemic infection occurs, the disease is advanced, and pathological lesions are assumed but we did not see any, reinforcing the idea that more studies are needed to fully apprehend the pathophysiology of *MAP* occurrence in carnivores and for which *MAP* isolation will be crucial. Although *MAP* can infect many wildlife species, evidence of clinical disease is more frequent among wild ruminants and lagomorphs^14,26^, while in predators and scavengers the intestinal and lymphoid tissues, if any, only have minor histopathological changes^18,86,87^.

*MAP* has been isolated from clinically healthy domestic and wild carnivores, but also from animals with diffuse microscopic lesions, for example red foxes in Scotland^18^, and sometimes dogs^88^, with clinical signs compatible with chronic gastrointestinal disease or lymphadenitis. Still, inflammatory lesion patterns are usually less extensive in carnivores than in ruminants. Pathological findings in red foxes and mongooses associated to *MAP* presence were reported by Matos *et al*.^38^, who described macroscopic lesions in mesenteric lymph nodes. Bacilli isolation and/or DNA detection from several anatomic regions, including the brain of foxes and stone marten, were also reported by the same authors, suggesting disseminated infection. In contrast, we did not find any macroscopic lesions nor pathological evidence of generalized infection, even in specimens collected in the same study region. Thus, our results rather suggest that *MAP*-exposed carnivores were either sampled during a preclinical phase or may be able to control pathogen multiplication and limit inflammatory responses, lessening tissue damage in an eventual life-long infection. The apparent high proportion of *MAP* IS*900*-positive red fox and mongoose found during this study, concurrent with the apparent lack of gastro-enteric lesions and confirmation of IS*900* shedding through feces, strongly suggests the presence of subclinical carriers that may occasionally shed bacteria. Although signs of true infection could not be confirmed and since detection of *MAP* IS*900* in feces does not imply the microorganism is viable, at this stage we can only speculate that mongoose and fox may serve as source of infection to other animals and contribute for environmental contamination. *MAP* contagion through the environment is epidemiologically relevant, especially when dealing with infection-resistant competitor species, which appears to be the case of red fox and mongoose, as these may potentially influence infection dynamics in susceptible hosts, alleviating or aggravating downstream effects such as prevalence rates and density or range of susceptible populations. Establishment of insidious pathogens in already fragile communities may thereafter compromise viability of natural populations.

## Concluding Remarks

Paratuberculosis has a major impact in animal health, being associated with important economic losses when species of commercial interest or species under costly conservation programs are affected. Results from this work give strong indications that *MAP* circulates in wildlife species from specific geographic regions of mainland Portugal but further work is required to obtain an isolate of *MAP* that proves beyond any doubt that *MAP* is present in this wildlife population. At this time point, we cannot discard the possibility of having detected a non-culturable microorganism carrying IS*900*. Although directionality of transmission cannot be inferred at this stage, our data supports the findings from previous reports of *MAP* isolation in carnivores from Portugal, adding to the increasing body of evidence of wilderness exposure to *MAP* and possibly to subclinical *MAP* shedders. Environmental contamination as an indirect transmission path may be likely, which suggests that *MAP* ecology and resilience outside natural hosts, as well as sustained transmission in carnivores, may be dependent on abiotic factors (e.g. climate). Prolonged survival of *MAP* in the environment enacts risks to grazing livestock, to paratuberculosis-infected adjacent farms, and also at the livestock-wildlife interface.

Suspicion for the ecological spread of *MAP* reinforces the need for increased surveillance and action. Quantitative information on the prevalence and concentration of *MAP* in putative contaminated sources of exposure is required. Although transmission intervals and paths tend to be uncertain for slow-growing and slow-evolving bacteria such as *MAP*, supplementary sampling efforts should be carried out in the future in order to include more wild specimens from the less represented species and to reach more balanced communities across the Iberian territory. Also, optimization of sample storage (−80 °C) to retain *MAP* viability and optimization of culture procedures to enable bacterial isolation in future studies should allow quantification of the extent and directionality of *MAP* transmission between livestock and wild species and amongst the latter. Whole genome sequencing following culture of *MAP* is to be recommended to confirm transmission between wildlife and livestock. Finally, additional studies are needed to identify landscape-specific risk factors, particularly across environmental gradients. This information is critical to understanding the risk of exposure under a climate change scenario and to decreasing the occurrence of new infections and the prevalence of existing ones.

## Supplementary information

Supplementary Tables and Figures.

## Data Availability

The authors declare that all data enclosed in the manuscript will be made available to the community.
