# Supplementary material for: Ecological drivers of Mycobacterium avium subsp. paratuberculosis detection in mongoose (Herpestes ichneumon) using IS900 as proxy
Source: Sci Rep. 2020 Jan 21;10:860. doi: 10.1038/s41598-020-57679-3 (PMC6972914; doi:10.1038/s41598-020-57679-3)
Supplement: Supplementary file 1 — Supplementary Tables and Figures. [file 41598_2020_57679_MOESM1_ESM.pdf]

**Ecological drivers of *Mycobacterium avium* subsp. *paratuberculosis* detection in mongoose (*Herpestes ichneumon*) using IS*900* as proxy**

Mónica V. Cunha [1,2,3], Luís Miguel Rosalino[2], Célia Leão[1], Victor Bandeira[4], Carlos Fonseca[4], Ana Botelho[1], Ana C. Reis[1,2,3]

[1]INIAV, IP- National Institute for Agrarian and Veterinary Research, Av. da República, Quinta do Marquês, 2780 -157 Oeiras, Portugal.

[2]Centre for Ecology, Evolution and Environmental Changes (cE3c), Faculdade de Ciências da Universidade de Lisboa, Campo Grande, 1749-016 Lisboa.

[3]Biosystems & Integrative Sciences Institute (BioISI), Faculdade de Ciências da Universidade de Lisboa, Campo Grande, 1749-016 Lisboa.

[4]CESAM & Departamento de Biologia, Universidade de Aveiro, Portugal

**Supplementary Table 1.** Family, species, number and type of animal specimens processed for IS*900* detection.

| **Family** | **Species** | **Total of animals** | **Total samples** | **Animals with both matrices analyzed** |
|---|---|---|---|---|
| ***Canidae*** **(*n*=40)** | Red fox (*Vulpes vulpes*) | 40 | *n*= 26 F; *n*=29 S | 16 |
| ***Mustelidae*** **(*n*=8)** | Stone marten (*Martes foina*) | 4 | *n*=3 F; *n*=3 S | 2 |
| | European badger (*Meles meles*) | 4 | *n*=2 F; *n*=4 S | 2 |
| ***Viverridae*** **(*n*=5)** | Common genet (*Genetta genetta*) | 5 | *n*=2 F; *n*=4 S | 1 |
| ***Herpestidae*** **(*n*=149)** | Egyptian mongoose (*Herpestes ichneumon*) | 149 | *n*=78 F; *n*=109 S | 37 |
| **Total** | | 202 | *n*=111 F; *n*=149 S | 58 |

Legend: F - feces, S - spleen.

**Supplementary Table 2.** Mathematical formula for the calculation of diversity indices and non-parametric estimators.

| | **Formula** |
|---|---|
| Shannon-Wiener (Help *et al.,* 1998) | $= -,$ |
| Simpson (Help *et al.,* 1998) | $D = 1 -$ |
| Berger-Parker (Help *et al.,* 1998) | $N\infty =$ |
| Chao 1 (Gotelli and Cowell, 2011) | $S_{tot} = S_{obs} + (a^2 / 2b)$ |
| Chao 2 (Gotelli and Cowell, 2011) | $S_{tot} = S_{obs} + (a^2 / 2b)$ |

Legend: $pi =$ proportional abundance of species $i$ in the sample; $p_1$ - proportional abundance of the most common species in the sample $S_{obs}$ – total of observed species in our sample; Chao 1: a-singleton and b-doubleton, singleton and doubleton refers to abundance data, being the singletons the communities with one individual and doubleton the communities with two individuals; Chao 2: a-unique and b-duplicate, unique and duplicate, refers to incidence data, being unique the communities with one species and duplicates the communities with two species.

**Supplementary Table 3.** Diversity indices values by sampled district and NUTS (Statistical terrestrial units).

| | Shannon-Wiener | Simpson | Berger-Parker |
|---|---|---|---|
| **Districts** | | | |
| Évora | 0,691 | 0,498 | 1,875 |
| Beja | 0,153 | 0,058 | 1,030 |
| Lisboa | 0,349 | 0,198 | 1,125 |
| Vila Real | 1,168 | 0,640 | 2 |
| Portalegre | 0,655 | 0,463 | 1,571 |
| Santarém | 0,947 | 0,570 | 1,778 |
| Coimbra | 0,537 | 0,277 | 1,188 |
| Castelo Branco | 0,730 | 0,418 | 1,364 |
| Guarda | 0,562 | 0,375 | 1,333 |
| Viseu | 0,600 | 0,314 | 1,222 |
| Aveiro | 1,099 | 0,667 | 3 |
| **NUTS** | | | |
| North | 1,168 | 0,640 | 2,000 |
| Center | 0,899 | 0,457 | 1,408 |
| Alentejo | 0,540 | 0,326 | 1,253 |
| Lisboa | 0,349 | 0,198 | 1,125 |

Legend: North - Viana do Castelo, Braga, Porto, Vila Real and Bragança; Center – Aveiro, Viseu, Guarda, Castelo Branco, Leiria, Santarém and Coimbra; Alentejo – Portalegre, Évora, Beja and Setúbal.

**Supplementary Table 4.** Demographic information of sampled and IS*900*-positive animals.

| Geographic location | No of tested animals | No of positive animals | Cause of death | Age class | Year |
|---|---|---|---|---|---|
| Évora | *n*=13 HI<br>*n*=16 VV | <br>*n*=1 VV | *n*=10 H; 3 RK<br>*n*=15 H; 1 RK | 2 juveniles; 11 adults<br>3 cubs; 1 juvenile; 2 sub-adulsts; 7 adults; 3 NA | 2010 (*n*=10); 2011 (*n*=3)<br>2011 (*n*=4); NA (*n*=12) |
| Beja | *n*=66 HI<br>*n*=1 VV<br>*n*=1 MF | *n*=5 HI | *n*=66 H<br>*n*=1 H<br>*n*=1 RK | 6 juveniles; 60 adults<br>1 adult<br>1 adult | 2010 (*n*=8); 2011 (*n*=42); 2013 (*n*=16)<br>2010 (*n*=1)<br>2010 (*n*=1) |
| Lisboa | *n*=8 HI<br>*n*=1 VV | *n*=1 HI | *n*=8 H<br>*n*=1 H | 1 juvenile; 7 adults<br>1 adult | 2009 (*n*=1); 2010 (*n*=7)<br>NA (*n*=1) |
| Vila Real | *n*=5 VV<br>*n*=1 MM<br>*n*=1 MF<br>*n*=3 GG | *n*=1 VV | *n*=1H; 4 RK<br>*n*=1 RK<br>*n*=1 RK<br>*n*=3 RK | 1 juvenile; 4 adults<br>1 adult<br>1 adult<br>3 NA | 2010 (*n*=1); 2011 (*n*=1); NA (*n*=3)<br>2011 (*n*=1)<br>2011 (*n*=1)<br>NA (*n*=3) |
| Portalegre | *n*=7 HI<br>*n*=4 VV | <br>*n*=1 VV | *n*=7 H<br>*n*=4 H | 1 juvenile; 6 adults<br>4 adults | 2008 (*n*=1); 2010 (*n*=3); 2011 (*n*=3)<br>2010 (*n*=4) |
| Santarém | *n*=9 HI<br>*n*=5 VV<br>*n*=2 MM | | *n*=9H<br>*n*=5 H<br>*n*= 2 RK | 1 juvenile; 8 adults<br>5 adults<br>1 cub; 1 adult | 2010 (*n*=2); 2011 (*n*=7)<br>2011 (*n*=4); NA (*n*=1)<br>2011 (*n*=2) |
| Coimbra | *n*=17 HI<br><br>*n*=2 VV<br>*n*=1 MF | | *n*=17 H<br><br>*n*=2 H<br>*n*=1 RK | 3 juveniles; 11 adults; 3 NA<br><br>2 adults<br>1 adult | 2008 (*n*=2); 2010 (*n*=2); 2011 (*n*=7); 2013 (*n*=3); 2014 (*n*=3)<br>2006 (*n*=1); 2010 (*n*=1)<br>NA (*n*=1) |
| Castelo Branco | *n*=11 HI<br><br>*n*=3 VV<br>*n*=1 MM | | *n*=10 H; 1 RK<br><br>*n*=3 RK<br>*n*=1 RK | 1 juvenile; 10 adults<br><br>3 adults<br>1 adult | 2009 (*n*=3); 2010 (*n*=6); 2011 (*n*=1); 2012 (*n*=1)<br>2010 (*n*=1); 2011 (*n*=2)<br>NA ((*n*=1) |
| Guarda | *n*=3 HI<br>*n*=1 VV | | *n*=2 H; 1 RK<br>*n*=1 RK | 1juvenile; 2 adults<br>1 adult | 2009 (*n*=2); 2011 (*n*=1)<br>2010 (*n*=1) |
| Leiria | *n*=1 HI | | *n*=1 H | 1 adult | 2011 (*n*=1) |
| Viseu | *n*=9 HI<br><br>*n*=1 VV<br>*n*=1 GG | *n*=1 HI<br><br><br>*n*=1 GG | *n*=9 H<br><br>*n*=1 H<br>*n*=1 RK | 9 adults<br><br>1 adult<br>1 juvenile | 2010 (*n*=2); 2011 (*n*=5); 2012 (*n*=1); 2013 (*n*=1)<br>2010 (*n*=1)<br>2009 (*n*=1) |
| Faro | *n*=5 HI | *n*=2 HI | *n*=4 H; 1 RK | 5 adults | 2011 (*n*=5) |
| Aveiro | *n*=1 VV<br>*n*=1 MF<br>*n*=1 GG | *n*=1 VV<br>*n*=1 MF | *n*=1 RK<br>*n*=1 RK<br>*n*=1 RK | 1 adult<br>1 adult<br>1 adult | 2011 (*n*=1)<br>2005 (*n*=1)<br>NA |
| Total | *n*=149 HI<br>*n*=40 VV<br>*n*=4 MF<br>*n*=4 MM<br>*n*=5 GG | *n*=9 HI<br>*n*=4 VV<br>*n*=1 MF<br><br>*n*=1 GG | *n*=173 H<br>*n*=29 RK | *n*=4 cubs<br>*n*=19 juveniles<br>*n*=2 sub-adult<br>*n*=168 adults<br>*n*=9 NA | 2005 (*n*=1)<br>2006 (*n*=1)<br>2008 (*n*=3)<br>2009 (*n*=7)<br>2010 (*n*=43)<br>2012 (*n*=2)<br>2011 (*n*=99)<br>2013 (*n*=20)<br>2014 (*n*=3)<br>NA (*n*=23) |

Legend- cause of death: H-hunted, RK-road-killed; NA - not available; VV: *Vulpes vulpes*; MF: Martes foina; MM: Meles meles; GG: Genetta genetta; HI: *Herpestes ichneumon*

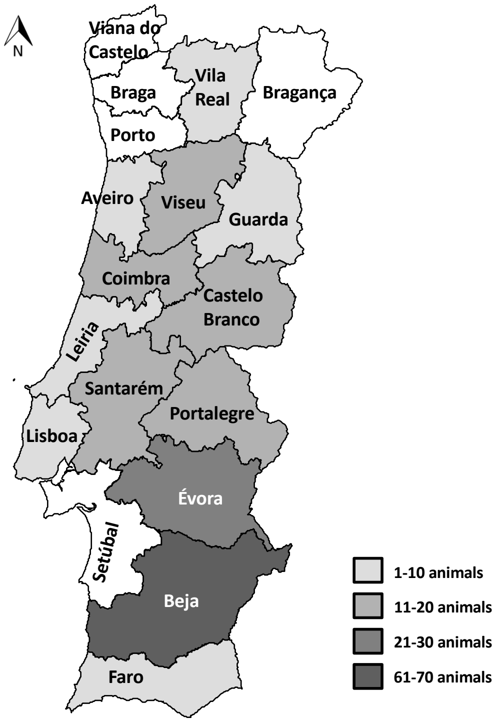


**Supplementary Figure 1.** The geographic distribution of the 202 animals under analysis across the districts of mainland Portugal is evidenced in grey scale.

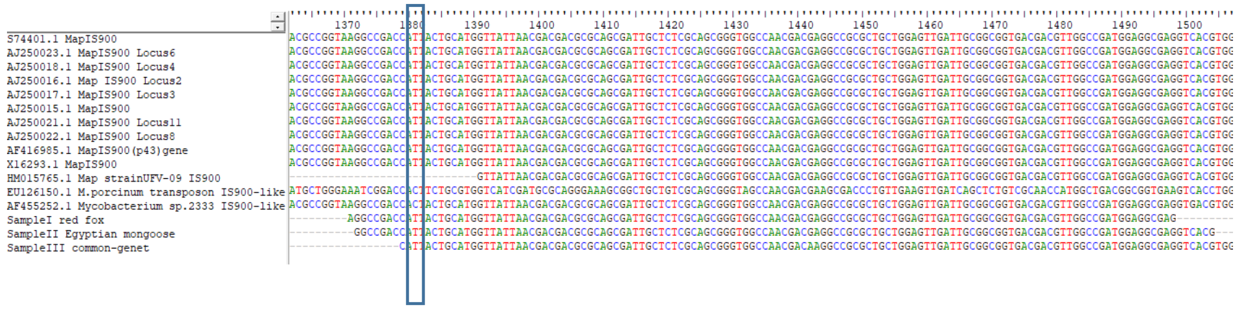


This mutation can distinguish both IS*900*-like sequences from all of the IS*900* sequences retrieved from NCBI, and from sequences obtained from sequencing of our samples by the amplification of a 224 bp fragment of IS*900* with external primers.

**Supplementary Figure 2.** Nucleotide sequence alignment of three 224 bp IS*900* amplicons from red fox, mongoose and genet (bottom), generated from the first PCR round, with 11 IS*900* sequences from 11 *MAP* strains genomes (Accession numbers AJ250023.1, AJ250018.1, AJ250016.1, AJ250017.1, AJ250015.1, AJ250021.1, AJ250022.1, S74401.1, AF416985.1, X16293.1 and HM015765.1) and two IS*900*-like sequences from *Mycobacterium* sp. 2333, accession number AF455252.1 and *Mycobacterium porcinum*, accession number EU126150.1. The SNP (T>C) that distinguishes IS*900*-like from MAP IS*900* is highlighted.

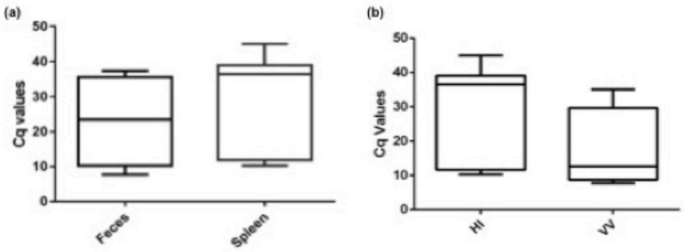


**Supplementary Figure 3.** Distribution of the Cq values obtained by semi-nested real-time IS*900* PCR for feces and spleen samples (a); and for Egyptian mongoose and red foxes samples (b). HI - *Herpestes ichneumon* and VV - *Vulpes vulpes*.
